# Supplementary material for: A set of multi-entry identification keys to African frugivorous flies (Diptera, Tephritidae)
Source: Zookeys. 2014 Jul 24;(428):97–108. doi: 10.3897/zookeys.428.7366 (PMC4143993; doi:10.3897/zookeys.428.7366)
Supplement: Supplementary material 6 — Key to Ceratitis [file zookeys-428-097-s006.zip › SF6_ZooKeys_key to Ceratitis/key/SF6_ZooKeys_key to Ceratitis/Media/Html/Ceratitis argenteostriata.htm]

Ceratitis (Pterandrus) argenteostriata, n


***Ceratitis*** **(*Pterandrus*) *argenteostriata* De Meyer & Freidberg**

Body
length: 4.42 (3.70-5.25) mm; wing length: 4.88 (4.30-5.45) mm.

Male

Head: Antenna yellow. First flagellomere 2-3
times as long as pedicel. Arista with short to moderately long rays; ventral rays
shorter and sparser than dorsal rays basally. Frons white, dorsal part more
yellow, rarely completely yellow; with short scattered setulae distinctly
darker than frons. Frontal setae well developed. Face whitish. Genal seta and
setulae dark, well developed.

Thorax: Postpronotal
lobe white anteriorly, with large dark area posterior to postpronotal seta.
Scutal pattern: ground color black, microtrichose areas with silvery shine,
forming two (pairs) distinct vittae; prescutellar white markings separated by
paler brownish area. Scapular setae dark, in one specimen medial scapular pale.
Scutellum basally yellowish white, without black spots, apically with three
joined black spots, extending to basal 0.33, weakly incised. Anepisternum on
ventral half brown or black; setulae pale.

Legs:
Yellow except where otherwise noted; setation typical for subgenus, mixed pale
and dark. Foreleg: femur anterodorsal half black, posteriorly largely black
except ventrobasally, with dispersed dark setulae along entire length but
without distinct bush, posterodorsal setulae longer; ventral setae dark.
Midleg: femur extensively darkened anteromedially, with silvery shine;
ventrally with black feathering along distal half, basally with few dispersed
long pale or dark setulae; tibia moderately broadened; largely blackish except
basally, silvery shine when viewed from certain angle; with black feathering
along distal 0.8 dorsally and 0.75 ventrally, with second row of short black
setulae anterior to ventral row. Hindleg: femur at apical 0.33 with long thin
setae dorsally and ventrally.

Wing:
Bands brown. Marginal band broadly separated from discal band around apex of
vein R_1_ and with large marginal hyaline spot around apex of vein R_2+3_;
cubital and medial bands free; crossvein R-M proximal to middle of discal cell.
Apex of vein R_1_ distal to level of crossvein R-M. Crossvein DM-Cu
oblique anterobasally.

Abdomen:
Predominantly yellow. Margin between tergites 1 and 2 largely brown. Tergites 2
and 4 with pale gray band along posterior half. Tergite 3 largely brown.
Tergites 4 and 5 with brown band along anterior half, sometimes interrupted in
middle. Posterior margin of tergite 5 narrowly brown. Setation typical for
subgenus. Male epandrium in lateral view with posterior lobe of lateral surstylus
slender and pointed.

Female

As
male except following characters: Antenna darker. Crossvein DM-Cu
oblique posterobasally. Legs
without feathering, setulae dark; femora darker brown on median part; forefemur
posteroventrally with dark setulae. Oviscape slightly shorter than preabdomen.
Aculeus at most five times as long as wide; tip pointed and lateral margin
straight.

(Description
after De Meyer & Freidberg, 2006)
